# Syndrome de Silver Russell: à propos de 3 cas et revue de la litterature

**DOI:** 10.11604/pamj.2013.14.91.1645

**Published:** 2013-03-08

**Authors:** Afaf Lamzouri, Ilham Ratbi, Abdelaziz Sefiani

**Affiliations:** 1 Centre de génomique humaine, Faculté de Médecine et Pharmacie, Université Mohammed V Souissi, Rabat, Maroc; 2 Département de génétique médicale, Institut National d’Hygiène, Rabat, Maroc

**Keywords:** Asymétrie, dysmorphie, empreinte parentale, retard staturo-pondéral, asymmetry, dysmorphia, parental imprinting, failure to thrive

## Abstract

Le syndrome de Silver Russell (SSR) est une maladie génétique rare. Sa prévalence est estimée à 1/100.000. Il s’agit d’une pathologie de l’empreinte parentale, caractérisée par une grande diversité phénotypique. Ses signes cliniques majeurs sont: un retard de croissance intra-utérin sévère, un retard staturo-pondéral post natal, une dysmorphie cranio-faciale particulière et une asymétrie des membres. Nous rapportons dans ce travail les observations de trois patients, qui présentent un retard staturo-pondéral, une dysmorphie faciale caractéristique du SSR et une hemihypertrophie corporelle. Nous discutons à travers ces cas les aspects cliniques et génétiques de ce rare syndrome.

## Introduction

Le syndrome de Silver Russell (SSR) est une maladie génétique rare dont la prévalence est estimée à 1/100.000. Elle est caractérisée par l’association d’un retard de croissance intra-utérin sévère, un retard staturo-pondéral post natal, une dysmorphie faciale particulière et une asymétrie des membres. Sur le plan génétique, le SSR est une pathologie de l’empreinte parentale. Il est sous la dépendance de plusieurs gènes soumis à empreinte, agissant par des mécanismes moléculaires différents.

Nous rapportons les observations de trois patients, adressés pour caryotype pour des motifs différents, et chez qui le diagnostic de SSR a été posé sur un faisceau de signes cliniques. Nous discutons à travers ces cas les aspects cliniques et les mécanismes moléculaires de ce rare syndrome.

## Patient et observation

**Figure 1:**
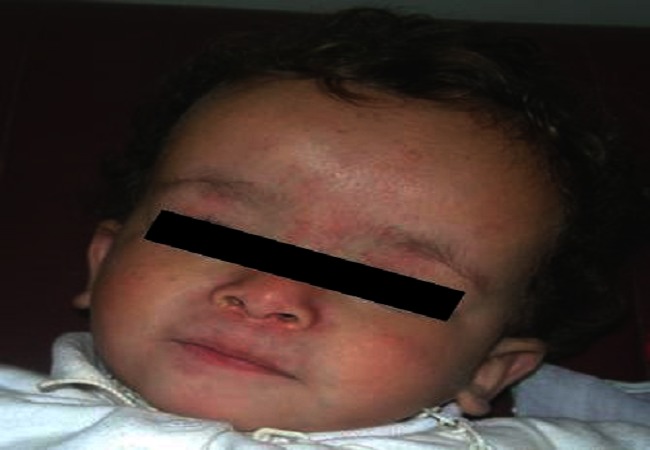
Dysmorphie faciale caractéristique du syndrome de Silver-Russell (Patient ≠ 1)

**Figure 2:**
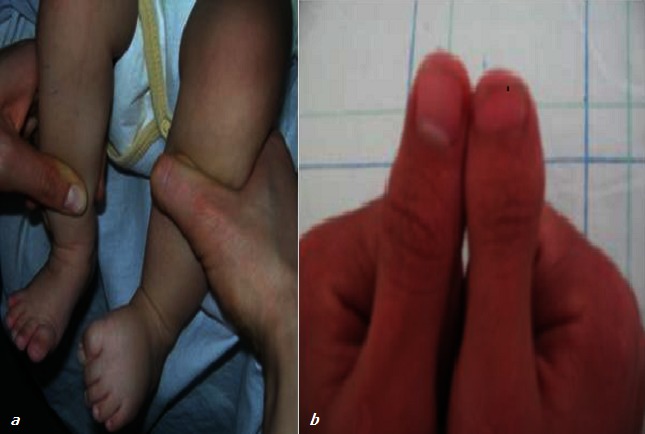
Asymétrie des membres (a: patient ≠1; b: patient ≠ 3)

Nous avons vu en consultation de génétique médicale trois patients chez lesquels nous avons retenu le diagnostic de SSR devant un faisceau de signes cliniques. Tous présentent au moins 3 signes majeurs du SSR: la dysmorphie faciale (Figure 1), le retard staturo-pondéral, l’asymétrie des membres (Figure 2), ainsi que plusieurs autres signes mineurs, résumés dans le Tableau 1.

**Tableau 1:** Caractéristiques cliniques et paracliniques des 3 patients atteints de SSR

	Patient ≠ 1	Patient ≠ 2	Patient ≠ 3
**Age**	1 an	2 ans	17 ans
**Motif de consultation**	Ambigüité sexuelle	Dysmorphie + RSP	RSP
**Sexe d’élevage**	Masculin	Féminin	Masculin
**Consanguinité**	-	+ (1^er^ degré)	-
**Accouchement**	A terme	A terme	A terme
**Hypotrophie néonatale**	+	+	ND
**DPM**	Normal	Retardé	Normal
**RSP**	-3DS	-4DS	-3DS
**PC**	Normal	Normal	Normal
Dysmorphie faciale du SSR*	+	+	+
**Asymétrie des membres**	+	+	+
**Signes mineurs**	Clinodactylie du 5^ème^ doigt droit	Difficultés alimentaires	Brachymétatarsie droite
	Transpiration excessive		Difficultés alimentaires
	Lèvres minces		Ectopie testiculaire
	Dents mal orientées.		
**Caryotype métaphasique**	46,XY	46 ,XX	46,XY
**Bilan thyroïdien**	Normal	Normal	Normal
**Dosage de l’hormone de croissance**	Non fait	Normal	Normal

**ND:** Non déterminé ; **DPM:** Développement psychomoteur ; **RSP:** Retard staturo-pondéral ; **PC:** Périmètre crânien ; Dysmorphie faciale du SSR*: Front large et bombé, petit visage triangulaire, micrognathie, sclérotiques bleutées et oreilles antéversées.

## Discussion

Le SSR, décrit pour la première fois en 1953 [1], est une maladie rare dont la prévalence est estimée à 1/100.000 [2]. Environ 400 cas ont été rapportés dans la littérature [3].

**Sur le plan clinique**: il existe une grande diversité phénotypique. Le SSR associe un retard de croissance débutant pendant la période anténatale à un aspect caractéristique du visage et une asymétrie des membres. Le retard de croissance intra-utérin est sévère alors que les enfants naissent habituellement à terme et la croissance post-natale reste insuffisante. Le déficit pondéral est souvent plus marqué que celui de la taille. Il existe également un retard de l’âge osseux et parfois un retard de fermeture de la fontanelle antérieure. Le volume du crâne est normal contrastant avec la taille donnant ainsi un aspect de pseudohydrocéphalie. Le front est large et bombant contrastant avec un petit visage triangulaire et une micrognathie. Les yeux paraissent grands avec des cils longs et des sclérotiques bleutées, et les oreilles sont grandes et antéversées. La bouche est large, avec des coins tombants, des lèvres minces et des anomalies dentaires. Une asymétrie (latérale et le plus souvent partielle) des membres, non évolutive, est présente dans 60% à 80% des cas, avec une clinodactylie des 5^èmes^ doigts [4]. La majorité de ces signes sont présents chez nos trois patients. Le développement psychomoteur est souvent normal mais, dans certains cas, il existe un retard des acquisitions motrices comme chez le patient ≠ 2. Une tendance à l’hypoglycémie en période néonatale, des troubles digestifs, une transpiration excessive et des taches cutanées café au lait sont des signes qui peuvent être parfois observés. Plus rarement, on peut trouver une syndactylie des orteils, des malformations génitales et des anomalies cardiaques et rénales [4].

Le diagnostic différentiel peut se poser devant toutes les causes environnementales et génétiques du retard staturo-pondéral pré et post natal. Mais un interrogatoire détaillé, un examen dysmorphologique et clinique complet, permettent en général de poser le diagnostic.

**Sur le plan génétique**: le SSR est une pathologie de l’empreinte parentale. C’est un phénomène physiologique qui concerne quelques dizaines de gènes, et qui conduit, dans des conditions normales, à l’expression d’une seule des deux copies parentales de chacun de ces gènes, soit la copie maternelle, soit la copie paternelle selon le gène considéré. Les gènes soumis à empreinte parentale jouent un rôle dans la croissance f’tale et post-natale.

Ce syndrome est sous la dépendance de plusieurs gènes soumis à empreinte:

- les gènes *H19 et IGF2* en 11p15 soumis respectivement à empreinte paternelle (inactifs sur le chromosome d’origine paternelle) et maternelle [4].
- le gène *GRB10* en 7p et le gène *MEST* en 7q soumis respectivement à empreinte paternelle et maternelle [5].

Il existe plusieurs mécanismes moléculaires responsables du SSR. Dans 7% à 10% des cas, une unidisomie parentale maternelle du chromosome 7 est mise en évidence [5]. Ce sont des sujets qui ont hérité de deux chromosomes 7 maternels sans contribution paternelle. Dans 35% à 50% des cas, une anomalie *épigénétique* de la région 11p15 est retrouvée [6]. C’est une anomalie qui ne modifie pas la séquence du gène mais l′organisation chromatinienne. Il s’agit d’une mutation de séquences appelées centres d’empreinte qui contrôlent la mise en place correcte de la méthylation dans les régions soumises à empreinte. La plupart des cas du SSR sont sporadiques. Quelques cas de récurrence intra-fratrie ont été rapportés [7] et une possible transmission parent-enfant a été signalée mais dans des cas qui ne semblent pas typiques [4].

Le conseil génétique dépend du mécanisme moléculaire sous-jacent. Le risque de récurrence est très faible en cas d’unidisomie parentale du chromosome 7; le conseil génétique est donc rassurant. Ce risque est plus élevé en cas de mutation du centre d’empreinte qui est transmise selon un mode mendélien [7]. Le traitement est symptomatique. L′administration d′hormone de croissance accélère la croissance et augmente la taille définitive, mais ne permet pas d′atteindre la taille cible. En dehors de la persistance d′une petite taille et d′une faible corpulence, le pronostic à long terme est bon [7].

## Conclusion

Le syndrome de Silver Russell est une maladie génétique liée à une anomalie de l’empreinte parentale. Le généticien joue un rôle important dans le diagnostic, qui est essentiellement clinique, et le conseil génétique de ce rare syndrome.
